# Long Working Hours and Emotional Well-Being in Korean Manufacturing Industry Employees

**DOI:** 10.1186/2052-4374-25-38

**Published:** 2013-12-05

**Authors:** Kyoung-Hye Lee, Jong-Eun Kim, Young-Ki Kim, Dong-Mug Kang, Myeong-Ja Yun, Shin-Goo Park, Jae-Seok Song, Sang-Gil Lee

**Affiliations:** 1 Department of Preventive and Occupational Medicine, School of Medicine, Pusan National University, Yangsan, South Korea; 2 Department of Occupational & Environmental Medicine, Pusan National University Yangsan Hospital Yangsan, South Korea; 3 Department of Occupational & Environmental Medicine, Inha University Collage of Medicine, Incheon, South Korea; 4 Kwandong University Collage of Medicine, Department Preventive medicine & Public health, Gangneung, South Korea; 5 Occupational Safety & Health Research Institute, KOSHA, Incheon, South Korea

**Keywords:** Long working hour, Emotional state, WHO (five) well-being index, Korean working condition survey

## Abstract

**Objectives:**

Korea is well known for its long work hours amongst employees. Because workers of the manufacturing industry are constantly exposed to extended work hours, this study was based on how long work hours affect their emotional well-being.

**Methods:**

The analysis was done using the secondary Korean Working Condition Survey (KWCS). Long work hours were defined to be more than 48 hours, and they were subcategorized into units of 52 hours and 60 hours. Based on the WHO (five) well-being index, emotional state was subdivided into three groups - reference group, low-mood group, and possible depression group- where 28 points and 50 points were division points, and two groups were compared at a time. Association between long work hours and emotional state was analyzed using binary and multinomial logistic regression analysis.

**Results:**

Working for extended working hours in the manufacturing industry showed a statistically significant increase (*t* test p < 0.001) in trend among the possible depression group when compared to the reference group and the low-mood group. When demographical characteristics, health behaviors, socioeconomic state, and work-related characteristics were fixed as controlled variables, as work hours increased the odds ratio of the possible depression group increased compared to the reference group, and especially the odds ratio was 2.73 times increased for work hours between 48–52 and 4.09 times increased for 60 hours or more and both were statistically significant. In comparing the low-mood group and possible depression group, as work hours increased the odds ratio increased to 1.73, 2.39, and 4.16 times, and all work hours from working 48–52 hours, 53–60 hours, and 60 hours or more were statistically significant. Multinomial logistic regression analysis also showed that among the reference group and possible group, the possible depression group was statistically significant as odds ratio increased to 2.94 times in working 53–60 hours, and 4.35 times in 60 hours or more.

**Conclusions:**

Long work hours have an adverse effect on emotional well-being. A more diversified research towards variables that affect long work hours and emotional well-being and how they interact with each other and their relationship to overall health is imperative.

## Introduction

Long working hours is one of the prominent characteristics of South Korea. According to 2010Organization for Economic Cooperation and Development(OECD) statistics, Korea had 45.9 hours per week, placing itself in second place out of all OECD countries following closely behind Turkey(49.3 hours per week) [1]. Moreover, the fifth European Working Condition Survey(EWCS) claimed that Korea had an additional 8.4 hours per week than the average 37.5 hours, which is the 2010 average weekly working hours of the European Union (EU) [2]. Long work hours are associated with its negative effects on overall health, such as poorly perceived general health, increased injury rates, increased reports of illnesses(myocardial infarction, hypertension, neck or musculoskeletal discomfort), increased mortality, and increased risk of premature birth in pregnancy [3]. A study conducted in Korea claims that increase in work hours were associated with increased severity of depression(as measured by Beck Depression Inventory(BDI)) and depressive symptomatology [4,5] and more specifically, working more than 60 hours per week was closely related to increase in suicidal thoughts [6]. However, the study supervised by Heo et al (2012) [4] concerning depression and length of work hours was limited to Labor Union members and reported results from a simple analysis done with a standard of 48 hours per week and thereby could not specifically isolate long work hours and its effects. Similarly, a study done by Kim et al (2013) [5] which was conducted by asking the question, ‘Have you ever experienced extreme feelings of sadness or despair that continuously impaired daily activities for more than two weeks in the past year?’ as a means of obtaining data did not prove to be a good indicator of overall emotional health. Similarly, Kim et al (2012) [5] could not deduce that suicidal thoughts are associated with long work hours due to inconsistencies in controlled variables such as temporary employment status and diverse occupations among subjects. Therefore, it is necessary to do research where depressive symptoms are included in emotional status and select a group of participants with a steady work schedule in order to observe how extended work hours affect emotional state.

Positive emotional states have the potential to espouse healthy perceptions, beliefs, and physical well-being. Not only do emotional states have the capacity to directly induce physiological responses, especially by influencing the immune system, they can also influence patterns of health behavior and affect progress of illness by either appropriately responding to health problems or eliciting social support through interpersonal relationships [7]. In order to appropriately evaluate mechanisms of emotional states, testing motor expressions of motor neuronsby using tools such as physiological arousal, Emotion Facial Action Coding System (EMFACS) and recognition of emotion in speech, or utilizing more subjective forms of testing such as PAD Emotion Scales, Self-Assessment Manikin (SAM), Geneva Emotions Wheel, and Product Emotion Measure Instrument v7.0 (PrEmo), is common [8]. However, such tests require a long-term commitment and have to be performed by a specialist, which is not applicable in studying a large-scale population. WHO (Five) Well-Being Index was initially developed to monitor emotional states of diabetic patients, and it is an effective tool that utilizes a short questionnaire to monitor emotional functioning of patients. Emotional well-being that is measured from the Index is an important measure of overall perceived quality of life, and it is a dependable screener for emotional function and depression [9]. Therefore, it is an appropriate mechanism for a study that requires a large-scale population.

One widely used tool is WCS (Working Condition Survey), a survey that is used to select a significant group of subjects from the working population and comprehend an economically active population and their work environment, which is used as a reference when deciding policies that increase the quality of work while pursuing improvement in productivity and increase in employment. EU is known for conducting this WCS every 5 years. Similarly, Korea Occupational Safety and Health Agency(KOSHA) has been conducting Korean Working Condition Survey(KWCS) to not only emulate EU’s WCS to analyze working conditions and reform working environment, but also in order to grasp causation of work-related risk factors in labor markets and provide baseline data concerning occupational safety and health policies that are appropriate for Korea. Risk factors associated with work environment including long work hours can be analyzed by examining studies representatives of the working population of a country, such as data from working condition survey.

Therefore, we conducted a study including regular employees of manufacturing industry who were chosen as subjects as they are representative of Koreans with relatively regular and set work schedules. WHO well-being index was used to control demographical characteristics, personal health behaviors, socioeconomic status, and work-related characteristics, and various studies were performed in order to understand an association between long work hours and emotional well-being.

## Materials and methods

### Data source

This study was based on the secondary Korean Working Condition Survey (KWCS) in 2010. The subjects of the study were economically active people at least 15 years old. The secondary KWCS was performed from June 20th, 2010 to October 10th, 2010. According to the Labor Standards Act, special working hours are included for industries such as transportation, sales and warehousing of goods, finance and insurance, film production and promotion of entertainment business, telecommunications, educational research and survey, advertising, medical services and sanitation, service, incineration and cleaning, and barbers. Those nominated by the presidential decree for public convenience and the characteristic of the task are also included as special working hours, and such industries may alter their working and non-working hours. Based on the Act, an economic sector was initially selected that is not granted a special working hour, which includes agriculture, forestry, fishing, mining, quarrying, and manufacturing industry. Excluding mining and quarrying as a possible candidate due to a lacking number of people (total 10), the manufacturing industry was chosen over agriculture, forestry, and fishing as a subject due to its stable work environment. Out of 10,019 laborers in this economic sector, those who work in the manufacturing industry were 1,293.

As stated in EWCS, the study was only limited to wage earners, excluding part-time hours which were categorized as weekly work hours less than 34 hours, and sole proprietorships, small businesses, and unpaid family workers were also excluded. Out of all 1,064 wage earners whose weekly work hours were longer than 34 hours, only regular employees out of day laborers, temporary employees, and regular employees were chosen in order to eliminate emotional fluctuation due to job insecurity. Regular employees are defined to have a minimum period of employment of more than 1 year or exclusive of such limitations. Regular employees of the manufacturing industry whose weekly work hours are over 34 hours and are subjects of the study were 993, weighted number of 3,336,970.

### Methods

Categorical variables such as demographic characteristics, individual health behaviors, and socioeconomic characteristics including gender, age, level of education, smoking, alcohol consumption, obesity, physical activity, and income were deduced. The sample was divided into 4 age groups: 15–29, 30–39, 40–49, 50 and over, and education level into three groups: middle school graduate, high school graduate, community college graduate or greater level of education. Smoking and alcohol consumption were also categorized into current smokers and high-risk drinkers by guidelines from the community health survey. Smokers were divided into quit smoking/have smoked in the past and currently smoking. Men who consumed at least 6 drinks per week or consumed alcohol at least twice a week and women who consumed at least 4 drinks per week or consumed alcohol at least twice a week were considered high-risk drinkers. Obesity was determined by questioning if there is a history of being diagnosed as being obese by a physician. Physical activity including sports, outdoor activities, cultural activities, and leisure activities, was categorized into whether an individual was physically active at least 3 times per week or less. Average monthly income was divided into categories of 1,000,000 won.

Company size was discerned by current number of employees, and they were categorized into 4 groups: less than 5, 5–49 50–299, 300 or more. Poor employment condition was thought to be expected from non-hiring companies and it was used to represent the working environment of the manufacturing industry. The questionnaire included a yes/no question in order for employees to answer if their schedules included shift work.

Work hours were classified so that 35–47 hours is a standard working hour, and 48 hours or more is considered an extended working hour, as categorized by EWCS [2]. Implementing the limitations of extended work hour of 52 hours by the Labor Standard Acts and chronic overwork index of 60 weekly work hours that results in cardiovascular disease and cerebrovascular disease as approved and enforced by revised ordinance Industrial Accident Compensation Insurance Act, extended working hours was subdivided into three groups: 48–52, 53–60, 61 or more.

The Well-being Index (ver.1998) [10] by WHO was utilized as the questionnaire concerning emotional state and well-being. The WHO Well-being Index is an excellent indicator of depression and overall emotional functioning by covering various emotions, from positive emotions (feeling content and relaxed), exuberance (being physical active and feeling refreshed in the morning), to general attentiveness and interest. Five questions were given:

1. I have felt cheerful and in good spirits.

2. I have felt calm and relaxed.

3. I have felt active and vigorous.

4. I woke up feeling fresh and rested.

5. My daily life has been filled with things that interest me.

Subjects in the study were to choose out of six different categories to answer these questions to indicate their emotional state in a past two-week period from: always, mostly, less than a week, sometimes, never. Raw scores of these answer range from 0 to 25, and weighted scores are from 0 to 100 by multiplying 4 to raw scores. Subjects with weighted score less than or equal to 28 are very likely to suffer from symptoms of depression and are encouraged to take further assessments to correctly diagnose such symptoms. Those with weighted score between 29–50 are considered to have low mood. Based on these two emotional indicatives provided by WHO (five) Well-Being Index, this study categorized those who scored between 51–100 as a reference group, those with 29–50 as low mood group, and rest of the subjects as possible depression group. It was shown in the preceding research that by non-parametric Mokken analysis, Loevinger coefficient of homogeneity was at least 0.5, which is a strong indicator of scalability and in turn supports the internal validity of WHO (five) Well-Being Index [11]. Analysis of the confidence interval of the variable utilized by the Working Condition Survey (WCS) showed that Cronbach’s α was 0.931, and the variables used in the study had Cronbach’s α of 0.927.

### Statistical analysis

**Figure 1:**
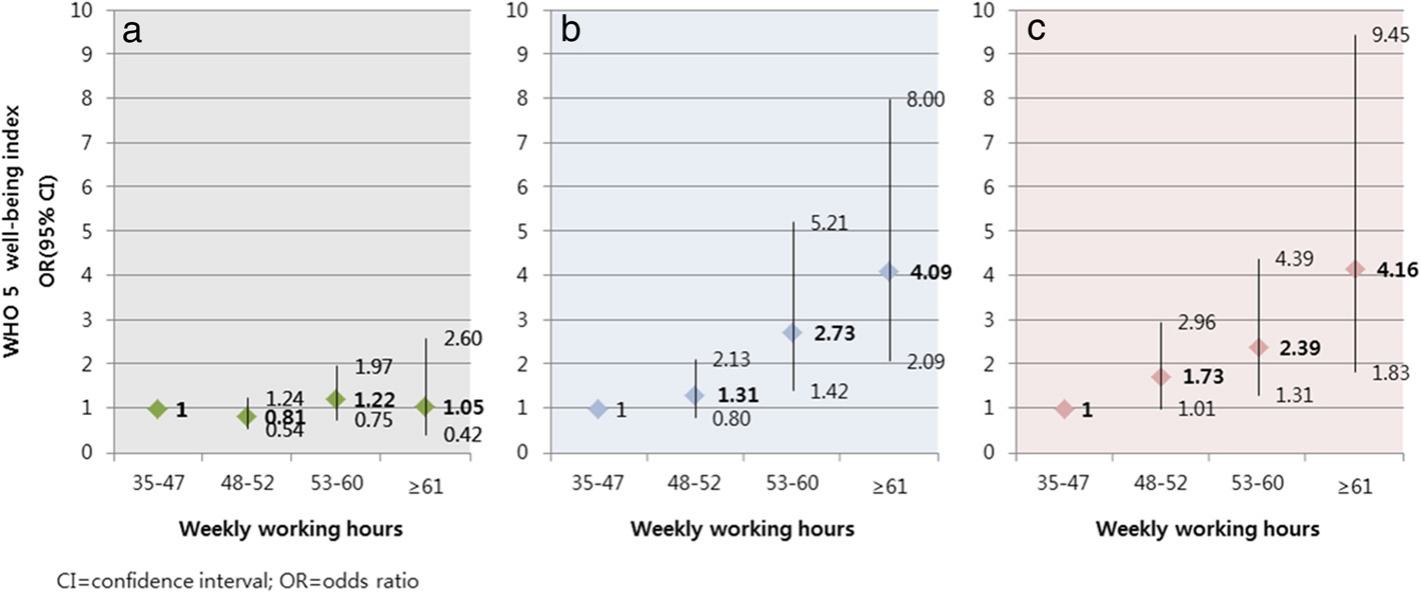
**Binary logistic regression analysis for emotional well-being due to working hour and risk factors.** Working hours adjusted for demographic factor (gender, age), health behaviors (smoking, alcohol drinking, diagnosed obesity, physical activity), socioeconomic state (education, income), and work characteristics (company size, shift work). **a)** comparison between reference group and low mood group, **b)** comparison between reference group and possible depression group, **c)** comparison between low mood group and possible depression group of WHO (five) well-being index.

WHO (five) Well-Being Index was subcategorized into three different groups by demographic characteristics, individual health behaviors, socioeconomic characteristics and work-related characteristics and compared for their differences. By applying two-step stratified PPS (Probability Proportional to Size) sampling when sampling a population, weighted values reflect the difference of extraction probability in each household within an enumeration district. Rao-Scott Chi-Square Test was used as the statistics test. To compare highest group, low mood group, and possible depression group from the WHO (five) Well-Being Index, multinomial logistic regression analysis was used, and by considering general characteristics (level of education, smoking, alcohol consumption, obesity, physical activity, income) and work-related characteristics (size of company, availability of shift work) as independent variables, odds ratio of adjusted work hour was calculated. Each odds ratio was then compared using binary logistic regression and the results are shown in Figure 1. By utilizing functions proc surveyfreq and proc surveylogistic from the SAS 9.2 program and taking weighted values into account, statistical analysis was executed according to stratified PPS (Probability Proportional to Size) sampling. Percentage values in descriptive statistics also reflected in weighted value. Estimates of population-based adjusted odds ratio and its 95% confidence interval were obtained through logistic regression analysis. The association between increase in work hours and variables was confirmed through Cochran-Armitage Trend Test.

## Results

### Employees of manufacturing industry and their general characteristics

Out of the subjects of the study who worked for more than 34 hours, male employees (77.5%) were predominant in the manufacturing industry compared to female employees (22.5%), and most were in their thirties (34.9%) and forties (29.4%). Majority of employees either graduated from high school (47.1%) or graduated from a community college or greater (46.1%). In relation to individual health behaviors, almost half of the subjects were current smokers (44.9%), and less number of people was high-risk drinkers (20.2%). Although only 3.4% of the subjects were considered obese, a surprising majority of them were physically inactive (84.6%). The greater number of employees earned between 1,000,000 and 2,000,000 won per month (33.1%).

Most manufacturing companies consisted of 5 to 49 employees (44.2%). Very few did shift works (14.1%). Most answered that they worked between 35 hours to 47 hours (51.5%), while 25.6% answered they worked between 48 to 51 hours, 15.9% between 53 to 60 hours, and 7.0% more than 60 hours. Regarding the WHO Well-Being index, the reference group turned out to be the largest where 52.4% scored between 51–100 points, while 23.3% scored between 29–50 points and was identified as a low-mood group and 17.2% scored 28 points or less and was identified as a possible depression group (Table 1).

**Table 1:** Study subject’s characteristics of Korean manufacturing industry on the second Korean working condition survey (N^*^ **= 993)**

**Variables**	**WHO (five) well-being index**												**p-value**^**†**^		
	**Reference group (A)**				**Low mood group (B)**				**Possible depression group (C)**				**A:B**	**A:C**	**B:C**
	**Unweighted frequency**	**Weighted frequency**	**Percent (%)**	SE^**^	**Unweighted frequency**	**Weighted frequency**	**Percent (%)**	SE^**^	**Unweighted frequency**	**Weighted frequency**	**Percent (%)**	SE^**^			
**Total**	**561**	**1,983,111**	**52.4**	**2.00**	**247**	**779,075**	**23.3**	**1.67**	**185**	**574,932**	**17.2**	**1.85**			
Gender													0.303	0.509	0.303
Female	144	473,160	63.02	1.28	53	156,936	20.90	0.75	43	120,768	16.08	0.74			
Male	417	1,509,951	58.38	1.89	194	622,138	24.06	1.54	142	454,163	17.56	1.67			
Age													0.045	0.722	0.232
<30	89	426,902	64.10	1.60	25	111,244	16.70	0.76	30	127,837	19.20	0.94			
30 - 39	202	724,667	62.24	1.72	82	260,735	22.39	0.91	51	178,891	15.36	1.20			
40 - 49	170	533,161	54.37	1.35	97	283,466	28.91	1.00	64	163,919	16.72	0.69			
≥50	100	298,380	56.69	1.12	43	123,630	23.49	0.60	40	104,285	19.81	0.57			
Highest level of education													0.443	0.004	0.030
Below middle school	48	131,083	57.35	0.62	16	38,580	16.88	0.31	26	58,907	25.77	0.39			
Graduate high school	240	856,641	55.74	1.71	114	372,103	24.21	1.09	97	308,163	20.05	9.23			
Above college	273	995,387	63.33	1.79	117	368,391	23.44	1.25	62	207,862	13.23	1.03			
Smoking													0.410	0.249	0.611
Ex-smoker or never smoker	324	1,134,728	61.71	1.85	136	415,345	22.59	1.37	96	288,737	15.70	1.11			
Present smoker	237	848,383	56.62	1.59	111	363,729	24.28	1.09	89	286,195	19.10	1.36			
Alcohol drinking													0.178	0.780	0.266
No or social drinker	448	1,562,632	58.69	2.07	210	650,762	24.44	1.60	147	449,324	16.87	1.35			
High risk drinker	113	420,479	62.35	1.23	37	128,313	19.03	0.68	38	125,608	18.63	0.98			
Diagnosed obesity													0.115	0.100	0.836
No	540	1,894,969	58.79	2.06	242	763,348	23.68	1.66	181	564,938	17.53	1.84			
Yes	21	88,142	77.41	0.68	5	15,726	13.81	0.23	4	9,994	8.78	0.15			
Physical activity^***^													0.005	0.010	0.952
No or not enough	455	1,608,080	56.87	2.03	223	702,449	24.84	1.59	167	517,283	18.29	1.72			
More than 3 days a week	106	375,031	73.64	1.22	24	76,626	15.05	0.55	18	57,649	11.32	0.43			
Average net monthly income (Korean Won, \^§^)													0.687	0.235	0.035
< 1,000,000	69	258,545	61.28	1.18	29	127,427	30.20	1.03	14	35,967	8.52	0.31			
1,000,000 ~ 2,000,000	192	654,230	59.28	1.59	75	228,454	20.70	0.83	71	221,003	20.02	1.01			
2,000,000 ~ 3,000,000	170	583,216	58.29	1.55	80	235,588	23.55	0.88	60	181,777	18.17	0.90			
≥3,000,000	130	487,119	60.07	1.50	63	187,604	23.14	0.85	40	136,184	16.79	0.97			
Company size(no. of employee)													0.107	0.216	0.411
<5	41	130,830	62.86	0.69	16	54,107	26.00	0.45	7	23,195	11.14	0.29			
5-49	234	808,088	56.19	1.80	119	378,464	26.32	1.20	86	251,525	17.49	0.93			
50-299	162	554,311	66.43	1.57	51	148,754	17.83	0.68	45	131,302	15.74	0.70			
≥300	110	436,026	56.34	1.75	53	172,717	22.32	1.03	45	165,113	21.34	1.29			
Weekly working hours													0.451	<0.001	<0.001
													(0.373)^‡^	(<0.001)^‡^	(<0.001)^‡^
Reference time(35–47)	297	1,903,463	63.62	1.92	126	423,350	24.63	1.44	70	201,988	11.75	0.82			
Long time1(48 ~ 52)	166	530,469	62.07	1.38	58	179,965	21.06	0.77	46	144,201	16.87	0.77			
Long time2(53–60)	71	254,940	47.97	1.03	51	134,215	25.26	0.57	43	142,269	26.77	0.89			
Long time3(≥61)	27	104,238	44.88	0.66	12	41,545	17.89	0.41	26	86,474	37.23	0.59			
Shift work													0.502	0.948	0.770
No	491	1,702,184	59.59	1.97	209	659,455	23.09	1.60	159	494,877	17.32	1.73			
Yes	70	280,927	58.45	1.14	38	119,620	24.89	0.63	26	80,055	16.66	0.72			

*unweighted case number of workers, **standard error, ***physical activity: sports, outdoor exercise, cultural activity, leisure activity, etc. †p-value by Rao Scott *X*2 test ‡p-value by Cochran- Armitage trend test §1USD($) = 1134.8KRW(\)(2010 Survey statistics by currency exchange rates, Ministry of Strategy and Finance, Korea).

### Characteristics of subjects and their emotional well-being state

Comparison between work hours of the reference group and low-mood group showed that they were not statistically significant (p = 0.451), nor was there a statistically significant trend (p = 0.373). However, a comparison between the low-mood group and possible depression group showed that a relationship between age and physical activity were statistically significant (p = 0.045, p = 0.005).

Both the relationship between working hours of the reference group and possible depression group was statistically significant (p < 0.001) and their trend was also statistically significant (p < 0.001). Moreover, education level and physical activity between the reference group and possible depression group showed that they were statistically significant (p = 0.004, p = 0.010).

Both the relationship between working hours of the low-mood group and possible depression group was statistically significant (p < 0.001) and their trend was also statistically significant (p < 0.001). Similarly, education level and income of low-mood group and possible depression group had similar results of a statistically significant trend (p = 0.030, 0.035) (Table 1).

### Binary logistic regression analysis of work hours and emotional well-being

Binary logistic regression analysis was performed on general characteristics (gender, age, socioeconomic status (level of education, income), individual health behaviors (smoking, alcohol consumption, obesity, physical activity) and work-related characteristics (size of company, availability of shift work).

In comparing reference group and low-mood group, change in emotions due to long work-hours was not observed. However, in comparison of reference group and possible depression group, increase of odds ratio regarding length of work time was observed in the possible depression group and was statistically significant; odds ratio of employees who worked between 53–60 hours and 60 hours or more each increased by 2.73 (95% confidence interval 1.42-5.21) and by 4.09 (95% confidence interval 2.09-8.40) and was both statistically significant. In comparing low-mood group and possible depression group, a statistically significant increase in odds ratio was observed according to length of work time, and odds ratio in the 48–52 hours, 53–60 hours, and 60 hours or more group was all statistically significant (Figure 1).

### Multinomial logistic regression analysis of work hours and emotional well-being

Multinomial logistic regression analysis was performed on general characteristics (gender, age, socioeconomic state (level of education, income), individual health behaviors (smoking, alcohol consumption, obesity, physical activity) and work-related characteristics (size of company, availability of shift work) of reference group, low-mood group, and possible depression group.

Change in emotional state due to long work hours was not observed when the reference group and low-mood group were compared. However, odds ratio of the possible depression group increased and was statistically significant as work hours grew longer when compared with the reference group; in long work hour intervals of 53–60 hours and 60 hours or more, each odds ratio in comparison to reference group was 2.93 (95% confidence interval 1.54-5.59) and 4.35 (95% confidence interval 2.18-8.16) (Table 2).

**Table 2:** Relation of emotional well-being with working hours and risk factors

**Variables**	**WHO (five) well-being index**^**†**^			
	**Low mood group**		**Possible depression group**	
	OR^*^	95% CI^**^	OR^*^	95% CI^**^
Weekly working hours				
Reference time(35–47)	1		1	
Long time1(48 ~ 52)	0.82	0.53 - 1.26	1.36	0.82 - 2.26
Long time2(53–60)	1.26	0.78 - 2.05	2.94	1.54 - 5.59
Long time3(≥61)	1.03	0.41 - 2.58	4.35	2.18 - 8.67
Gender				
Female	1		1	
Male	1.14	0.63 - 2.04	1.12	0.64 - 1.96
Age				
<30	1		1	
30 - 39	1.27	0.63 - 2.57	0.78	0.41 - 1.47
40 - 49	1.87	0.96 - 3.65	1.00	0.49 - 2.04
≥50	1.69	0.79 - 3.64	0.97	0.42 - 2.24
Level of education				
Above college	1		1	
Graduate high school	1.04	0.69 - 1.56	1.50	0.94 - 2.39
Below middle school	0.52	0.22 - 1.21	2.01	0.86 - 4.72
Smoking				
Ex-smoker or never smoker	1		1	
Present smoker	1.20	0.78 - 1.83	1.19	0.77 - 1.85
Alcohol drinking				
No or social drinker	1		1	
High risk drinker	0.67	0.41 - 1.12	1.08	0.65 - 1.81
Diagnosed obesity				
No	1		1	
Yes	0.37	0.11 - 1.27	0.29	0.07 - 1.25
Physical activity^††^				
More than 3 days a week	1		1	
No or not enough	1.97	1.12 - 3.45	1.79	0.99 - 3.24
Average net monthly income(Korean Won)				
≥3,000,000	1		1	
2,000,000-2,999,999	1.06	0.63 - 1.78	0.99	0.52 - 1.9
1,000,000 ~ 1,999,999	1.11	0.61 - 2.03	1.09	0.53 - 2.24
< 1,000,000	1.53	0.74 - 3.15	0.45	0.19 - 1.10
Company size(no. of employee)				
≥300	1		1	
50-299	0.60	0.33 - 1.08	0.56	0.27 - 1.17
5-49	1.04	0.62 - 1.75	0.61	0.31 - 1.22
<5	0.97	0.40 - 2.34	0.30	0.10 - 0.96
Shift work				
No	1		1	
Yes	1.16	0.68 - 1.98	1.36	0.82 - 2.26

^*^Odds ratio in multinomial logistic regression analysis by proc surveylogistic, ^**^confidence interval in multinomial logistic regression analysis, ^†^reference group: who (five) well-being index score from 51 to 100, ^††^physical activity: sports, outdoor exercise, cultural activity, leisure activity, etc.

## Discussion

This study observed the emotional well-being associated with work hours by utilizing WHO (five) well-being index. A trend test between the possible depression group that scored between 0–28 and the other two groups showed that when work hours increased, possibility of developing depression also increased. Moreover, odds ratio of the possible depression group increased as work hours grew longer compared to the reference group even as general characteristics and work-related characteristics were controlled as variable. Putting sleep cycle and imbalance between work and life in perspective, long work hours are likely to cause negative impact on emotional well-being. Chances to be develop social support such as physical activity and relationships outside the workplace decreases as work hours become longer. Of the sample that EWCS reported, only 14% was considered to be working long hours of 48 hours or more, which is significantly different compared to the 47.9% from this study. The previous study warns and calls for awareness of health problems that arises from imbalance between work and life, intensity of labor and increased exposure to danger related to long work hours [2], and Korea is predicted to have more health and social problems compared to EU due to a higher percentage of employees with long work hours.

Weekly work hours of an average manufacturing industry laborer was 47.1 hours in this study, while other studies such as Survey on Labor Conditions by Type of Employment [12] and Korean Labor and Income Panel Study (KLIPS) [13] each reported slightly different results of 46.1 hours and 43.2 hours for manufacturing employees in 2010. The survey on labor conditions by type of employment only focused on regular employees who worked at companies with 5 or more employees, while KLIPS focused on same sized companies as reported by the employer. This study includes smaller sized companies (less than 5 employees), unpaid rest breaks and meal periods and depends on self-report methods so discrepancies exist compared to official government statistics on work hours.

Emotional state is known to be associated with gender and age. While a notable discrepancy was observed between reference group and low-mood group, results of binary and multinomial logistic regression analysis showed that general characteristics and work-related characteristics were not statistically significant. It should be noted because females tend to express emotions more often, experience greater intensity of emotions and heightened sense of physiological awareness, it is perceived that gender difference in emotions exist [14,15]. It is possible that this study does not reflect such a gender difference because females may respond more positively to job security [16]. Moreover, even though it is well known that an emotional state is affected by age, as seen by observations of higher levels of both positive and negative emotional states during adolescence and senescence [17], a slight disparity was observed very likely due to underestimation of health worker effect [18].

A clear relationship between health behavior and emotional well-being was not observed in this study. Previous studies showed an association between employment status and change in health behaviors such as consumption of alcohol, weight and physical activity (excluding smoking), which is a good example of how stress due to change in employment status may cause changes in health behavior [19]. Similarly, this study took health behaviors into account such as smoking consumption of alcohol, weight and physical activity and studied their association, and both low-mood group and possible depression group showed a significant association. It is predicted that they are interrelated, where physical activity increases positive emotions and suppresses negative emotions, and negative emotions suppresses promotion of healthy behavior [5].

There are several limitations to this study. First, causal relationships are hard to be defined from a cross sectional study based on questionnaires. Not only do limitations exist as a survey to represent the Korean population, it is also a cross sectional study and difficult to say that emotional well-being has influenced work hours. Therefore it is probable that work hours and change in emotional well-being are in a causal relationship, and it is an issue that needs a confirmation through a Cohort study. This study has included various confounding variables in order to overcome such challenges; effects of shift work on health (based on previous studies) were included in the analysis and work-related characteristics (income, company size) were included as much as possible to interpret the relationship between long work hours and negative emotional state.

Secondly, there is a possibility that observational errors exist because KWCS measures work hours through a detailed questionnaire. Work hours tended to have a disparity compared to other well-known sources, and it is perceived to be caused by misunderstanding of terminology related to hours and difference in sampling. However, because the study employed the same questionnaire, it is thought that a questionnaire based work hour survey does not affect the direction or strength of association with emotional well-being.

Third, the WHO well-being index was used to diagnose a specific emotional state, but a more diverse approach is necessary in order to identify different emotional states. Even though the WHO well-being Index data is sufficient in measuring emotional functioning or diagnosing depression, it is not an applicable in correctly distinguishing an emotionate state. It is necessary that a research be conducted where an emotional state is subdivided into positive and negative emotions, or emotional states and traits. The WHO Well-Being Index is a survey based primarily on positive emotions. However, the basal ganglia and amygdala are each known to be closely involved with positive and negative emotions [20,21], so a classification between two emotional activities and a more extensive research on negative emotions are crucial. It is also possible to use a different approach where emotional state is subdivided into state emotion, a type emotion that one acquires from specific incidents, or trait emotion, a type of emotion that is constantly innate in every individual. State of emotions can be differentiated by using the Intensity and Time Affect Scale (ITAS) [22] to categorize emotional states by survey or by observing changes in emotions during elapsed time through journal entries [23]. However, aforementioned surveys are complicated, have many provisions, and require a significant amount of time and effort, which is more applicable for a smaller sized sample. It is more suitable to utilize the WHO (five) well-being index in monitoring emotional functioning, emotional well-being, possibility of developing depression, and the continual progress in a larger sample study.

The resulting work hours from this study shows an association between long work hours and emotional well-being. This result is similar to previous studies conducted in Korea where working 48 hours or more showed increase in level of depression (measured by Beck Depression Inventory (BDI)) [4] and working 60 hours or more showed increase in depressive symptoms [5] and suicidal tendencies [6]. Based on these results, conducting additional researches on variations of emotional well-being, health problems associated with this, health behaviors and progress in illnesses that arise from altering work hours is required. Furthermore, it would be necessary to subcategorize a long time frame in order to find the accurate initiation point where physical and emotional changes related to working long hours occur. This study has taken shift work into account in relation to work hours, but additional analysis of variables that might affect the result, such as work days, late night shifts, working all day long, working during the holidays, and flexibility of work schedule, is imperative.

This study was adequate in establishing an association between long work hours and emotional well-being by studying employees of manufacturing industry. Results show that with controlled variables, a clear association between long work hours and possibility of developing depression and emotional well-being was observed, as seen by trends and graphs. Not only are emotional states meaningful as a result by themselves, they also have a capacity to affect overall health and be used as a reference to indicate the current health of an individual [11]. Additional approaches concerning balance between work and life, sleep cycle, interpersonal relationships, negative effects on promotion of health, perception of health, behavior, occurrence of illness, and occurrence of natural disasters are necessary in order to create an even more diverse approach to emotional states and analyze how long work hours affect one’s health.

## Competing interests

The authors declare that they have no competing interests.

## Authors’ contributions

KHL, JEK, YGK, DMG and SGP designed the research. KHL, MJY and SGL collected the data and performed the statistical analysis. KHL, MJY, JEK, and DMG interpreted the data and wrote the manuscript. All authors read and approved the final manuscript.

## References

[B1] OECD OECD statistics Available: http://stats.oecd.org [cited 3 February 2013]

[B2] Agnès PT Greet V van Houten G Maija LY Biletta I Cabrita J Niedhammer I5th European working condition survey 2012 Luxembourg: European Union p32 p37

[B3] van der Hulst M Long work hours and health Scand J Work Environ Health 2003 25 3 181 188 10.5271/sjweh.720 12828387

[B4] Heo HT Kim DW Lee JS Jo HA Jang SS Kim SY Kim IA An association between working schedules and depression in public sector employees Korean J Occup Environ Med 2012 25 4 347 355 korean

[B5] Kim I Kim H Lim S Lee M Bahk J June KJ Kim S Chang WJ Working hours and depressive symptomatology among full-time employees: results from the fourt Korean national health and nutrition examination survey (2007–2009) Scand J Work Environ Health 2013 10.5271/sjweh.3356. [Epub ahead of print] 10.5271/sjweh.3356 23503616

[B6] Kim KU Park SG Kim HC Lim JH Lee SJ Jeon SH Huh YS Association between long working hours and suicidal ideation Korean J Occup Environ Med 2012 25 4 339 346 korean

[B7] Salovey P Rothman AJ Detweiler JB Steward WT Emotional states and physical healthy Am Phychol 2000 25 1 110 121 10.1037//0003-066x.55.1.110 11392855

[B8] Coan JA Allen JJB Handbook of emotion elicitation and assessment. Series in affective science 2007 New York: Oxford University Press 91 297

[B9] Snoek F WHO (five) well-being index 2013 Available: http://www.dawnstudy.com

[B10] World Health Organization WHO (five) well-being index (1998 version) 2013 Available: http://www.cure4you.dk/354/WHO-5_English.pdf

[B11] Bech P Measuring the dimension of psychological general well-being by the WHO-5 119 Qual Life 2004 25 15 16

[B12] Labor Market Analysis Division Survey report on labor conditions by employment type 2011 Seoul: Ministry of Employment and Labor pp 22 pp 28 korean

[B13] Labor Market Analysis Division 2012 Korean labor and income panel study 2011 Seoul: Ministry of Employment and Labor pp 3 pp 6 korean

[B14] King AM Gorden AH Sex differences in emotion: expression, experience, and physiology J Pers Soc Psychol 1998 25 3 686 703 9523412 10.1037//0022-3514.74.3.686

[B15] Gross JJ John OP Mapping the domain of expressivity: multimethod evidence for a hierarchical model J Pers Soc Psychol 1998 25 1 170 191 9457781 10.1037//0022-3514.74.1.170

[B16] Cox T Leka S Ivanov I Kortum E Work, employment and mental health in europe Work Stress 2004 25 2 179 185

[B17] Richard AF Carol LM Gender and age stereotypes of emotionality Pers Soc Psychol Bull 1991 25 5 532 540 10.1177/0146167291175008

[B18] Lea CS Hertz-Picciotto I Andersen A Chang-Claude J Olsen JH Pesatori AC Teppo L Wester-holm P Winter PD Boffetta P Gender differences in the healthy worker effect among synthetic vitreous fiber workers Am J Epidemiol 1999 25 10 1099 1106 10.1093/oxfordjournals.aje.a009935 10568626

[B19] Marianna V Mika K Matti J Pekka V Marko E Jussi V Temporary employment and health: a review Int J Epidemiol 2005 25 610 622 15737968 10.1093/ije/dyi024

[B20] Adam KA Elizabeth AP Is the human amygdala critical for the subjective experience of emotion? Evidence of intact dispositional affect in patients with amygdala lesions J Cogn Neurosci 2002 25 5 709 720 10.1162/08989290260138618 12167256

[B21] Ashby FG Isen AM Turken AU A neuropsychological theory of positive affect and its influence on cognition Psychol Rev 1999 25 3 529 550 10467897 10.1037/0033-295x.106.3.529

[B22] Diener E Larsen RJ Levine S Emmons RA Intensity and frequency: dimensions underlying positive and negative affect J Pers Soc Psychol 1985 25 5 1253 1265 3998989 10.1037//0022-3514.48.5.1253

[B23] Arther PB Howard MW Organizational behavior: affect in the workplace Annu Rec Psychol 2002 25 279 307 10.1146/annurev.psych.53.100901.135156 11752487

